# Supplementary material for: Complexome profiling of the Chlamydomonas psb28 mutant reveals TEF5 as an early PSII assembly factor
Source: Plant Cell. 2025 Mar 17;37(6):koaf055. doi: 10.1093/plcell/koaf055 (PMC12164590; doi:10.1093/plcell/koaf055)
Supplement: koaf055_Supplementary_Data [file koaf055_supplementary_data.zip › psb28_interactive.zip!/plots/intensity/qualityPlots/peptides/NUOS6_Cre03.g178250.t1.2.html]

### Cre03.g178250.t1.2

Trivial name: NUOS6  
SeqCoverage [median]: 29.20  
Mass: 16.28 kDa  
  
 

| Sequence | MissedCleavage | Mass [Da] | wtIntensity | muIntensity | Score |
| --- | --- | --- | --- | --- | --- |
| AADVVPTFDWK | 0 | 1247.62 | 1136.10 | 0.00 | 67.56 |
| ATSGLGLGDGIK | 0 | 1087.59 | 13398.83 | 13584.25 | 160.36 |
| GATPAGDLR | 0 | 856.44 | 14206.02 | 8125.58 | 89.75 |
| SPMEYINEVAPIK | 0 | 1489.75 | 6380.88 | 5519.52 | 163.12 |
|  |  |  | 35121.8 | 27229.4 |  |
